# Efficient full-path optical calculation of scalar and vector diffraction using the Bluestein method

**DOI:** 10.1038/s41377-020-00362-z

**Published:** 2020-07-13

**Authors:** Yanlei Hu, Zhongyu Wang, Xuewen Wang, Shengyun Ji, Chenchu Zhang, Jiawen Li, Wulin Zhu, Dong Wu, Jiaru Chu

**Affiliations:** 1 CAS Key Laboratory of Mechanical Behavior and Design of Materials, Key Laboratory of Precision Scientific Instrumentation of Anhui Higher Education Institutes, Department of Precision Machinery and Precision Instrumentation, University of Science and Technology of China, Hefei, 230026 China; 2 Department of Mechanical Engineering and Department of Civil and Environmental Engineering, Massachusetts Institute of Technology, Cambridge, MA 02139 USA; 3 State Key Laboratory of Advanced Technology for Materials Synthesis and Processing, International School of Materials Science and Engineering, Wuhan University of Technology, Wuhan, 430070 China; 4 Institute of Industry and Equipment Technology, Hefei University of Technology, Hefei, 230009 China

**Keywords:** Optics and photonics, Applied optics

## Abstract

Efficient calculation of the light diffraction in free space is of great significance for tracing electromagnetic field propagation and predicting the performance of optical systems such as microscopy, photolithography, and manipulation. However, existing calculation methods suffer from low computational efficiency and poor flexibility. Here, we present a fast and flexible calculation method for computing scalar and vector diffraction in the corresponding optical regimes using the Bluestein method. The computation time can be substantially reduced to the sub-second level, which is 10^5^ faster than that achieved by the direct integration approach (~hours level) and 10^2^ faster than that achieved by the fast Fourier transform method (~minutes level). The high efficiency facilitates the ultrafast evaluation of light propagation in diverse optical systems. Furthermore, the region of interest and the sampling numbers can be arbitrarily chosen, endowing the proposed method with superior flexibility. Based on these results, full-path calculation of a complex optical system is readily demonstrated and verified by experimental results, laying a foundation for real-time light field analysis for realistic optical implementation such as imaging, laser processing, and optical manipulation.

## Optics: Calculating diffraction effects in optical technologies

A fast and flexible procedure for evaluating the propagation of light in optical systems is achieved by calculating the diffraction of the light using a computational process called the Bluestein method. It yields information along the entire optical path length on both ‘scalar’ variations in the general magnitude of light waves and ‘vector’ – directional – variations. Researchers led by Jiawen Li and Dong Wu at the University of Science and Technology of China, developed the process and demonstrated that it can deliver results between 100 and 100,000 times faster than two existing alternative methods. Understanding the influence of diffraction in prevailing conditions is important for predicting the fine behaviuor of light waves. The new procedure should lead to greatly improved real-time analyses that will assist in microscopy, laser-based fabrication and optical manipulation technologies.

## Introduction

Diffraction is a classic optical phenomenon accounting for the propagation of light waves. The efficient calculation of light diffraction is of significant value toward the real-time prediction of light fields in microscopy^1^, laser fabrication^2–5^, and optical manipulation^6,7^. The diffraction of electromagnetic (EM) waves can be cataloged into scalar diffraction and vector diffraction according to the validation of different approximation conditions. Scalar diffraction considers only the scalar amplitude of one transverse component of either the electric or the magnetic field with certain simplifications and approximations^8^. Scalar diffraction can yield sufficiently accurate results if the diffracting aperture and observing distance are both far larger than a wavelength, which is most valid for optical systems with a low numerical aperture (NA). For high-NA optical systems, polarization effects play a paramount role near the focal spot, and thus, vector diffraction must be adopted for light field tracing^9–11^. Although mathematical expressions for optical diffractions have been presented authoritatively for ages, fundamental breakthroughs have rarely been achieved in diffraction computations. The direct integration method was first used to calculate both scalar and vector diffraction^12–14^. However, the point-by-point calculation fashion renders the computation extremely tedious and inefficient. Fast Fourier transform (FFT)-based algorithms have been developed to perform fast calculations of light diffraction^15–19^. However, these methods can generate only the light field distribution within a fixed region of interest (ROI) and sampling numbers (i.e., resolution) determined by the intrinsic characteristic of the Fourier transform (FT), lacking flexibility in computing the desired local distribution with variable sampling intervals. Therefore, the versatile computation of optical diffraction in an efficient and flexible fashion is highly demanded for wide applications.

In addition, scalar and vector diffractions are separately analyzed in conventional studies because different integral formulas are needed for each case. However, in most practical apparatuses, scalar and vector diffractions co-exist for different parts of the optical system. For example, in typical systems for optical microscopy, fabrication and manipulation, a monochromatic beam propagates over a long distance by passing optical elements such as focusing lenses, expanders, and collimators before entering an objective lens with a high NA. For the preceding part where the paraxial condition is valid, scalar diffraction is satisfactory for the light propagation evaluation. For the part behind the high-NA objective that meets the Debye approximation, vector diffraction is required for the accurate evaluation of the light propagation by taking into account each polarization component and non-paraxial propagation of light as well as apodization function of optical systems. Therefore, a facile and efficient method with the capacity for light propagation calculation along the entire optical path, which is termed full-path calculation, is highly desired for the comprehensive analysis of numerous realistic application scenarios.

Here, we propose an efficient full-path calculation method by exploring the mathematical similarities in scalar and vector diffraction. The scalar and vector diffraction are both expressed using the highly flexible Bluestein method. A fast light field evaluation over the entire optical path is achieved with arbitrarily defined ROIs and sampling numbers. This paper is organized as follows: first, the integral formulas for scalar and vector diffraction are revisited and deduced in FT forms. Second, the Bluestein method is utilized and reformed to completely supplant the FT in a more flexible fashion. Based on this, optical diffractions are evaluated with designated ROIs and sampling numbers. Third, representative examples are given for both scalar and vector diffraction to demonstrate the improvement in efficiency and flexibility. Finally, full-path light tracing of a laser holographic system is presented with unprecedented computation speed and agrees well with the experimental results, showcasing the superior ability of the Bluestein-based diffraction calculation. The proposed method holds great promise in the universal applications of optical microscopy, fabrication, and manipulation.

## Results

### Scalar and vector diffraction integral in the form of a Fourier transform

**Fig. 1:**
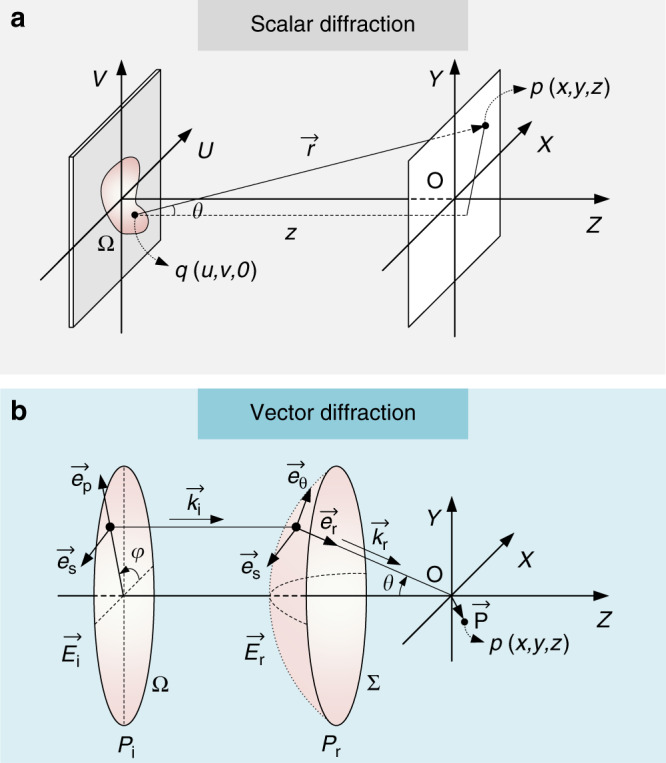
Illustrative diagrams of scalar and vector diffraction. **a** Geometry for scalar diffraction calculation. **b** Geometry for vector diffraction calculation

For scalar diffraction, as shown in Fig. 1a, the electric field at a point (*x, y, z*) in the Cartesian coordinates can be obtained based on the Huygens–Fresnel principle and expressed by the Rayleigh–Sommerfeld diffraction integral^20^:

1 $$E\left( {x,y,z} \right) = - \frac{i}{\lambda } {\iint_{\!\Omega}} {E_0\left( {u,v,0} \right) \times \frac{{\exp \left( {ikr} \right)}}{r} \times \cos \theta \;} dudv$$

where $r = \sqrt {\left( {x - u} \right)^2 + \left( {y - v} \right)^2 + z^2}$ is the distance between the source point and the observation point of interest. *k* = 2*π*/*λ* is the wavenumber. In the condition of the Fresnel approximation with a Fresnel number *F* ≥ 1, the third term and higher orders in the Taylor expression of *r* can be ignored, that is, $r \approx z + \frac{{\left( {x - u} \right)^2 + \left( {y - v} \right)^2}}{{2z}}$. In the denominator of Eq. (1), *r* can be further approximated with only the first term (*r* ≈ *z*). Moreover, the paraxial approximation ensures cos*θ* ≈ 1. In this way, the complex electric field can be described by the Fresnel diffraction integral:

2 $$E\left( {x,y,z} \right) = \frac{{\exp \left( {ikz} \right)}}{{i\lambda z}} {\iint_{\!\Omega}} {E_0\left( {u,v,0} \right) \times \exp \left\{ {\frac{{ik}}{{2z}}\left[ {\left( {x - u} \right)^2 + \left( {y - v} \right)^2} \right]} \right\}} dudv$$

which can be further rewritten as:

3 $$E\left( {x,y,z} \right) = \frac{{\exp \left( {ikz} \right) \times \exp \left( {ik\frac{{x^2 + y^2}}{{2z}}} \right)}}{{i\lambda z}}{\iint}_{\!\Omega} {E_0\left( {u,v,0} \right) \times \exp \left[ {\frac{{i\pi }}{{\lambda z}}\left( {u^2 + v^2} \right)} \right] \times {\mathrm{exp}}\left[ { - \frac{{2i\pi }}{{\lambda z}}\left( {xu + yv} \right)} \right]} dudv$$

Here, we define:

4 $$F_0 = \frac{{\exp \left( {ikz} \right) \times \exp \left( {ik\frac{{x^2 + y^2}}{{2z}}} \right)}}{{i\lambda z}}$$

5 $$F = \exp \left[ {\frac{{i\pi }}{{\lambda z}}\left( {u^2 + v^2} \right)} \right]$$

Therefore, the integral Eq. (3) can be expressed in terms of the two-dimensional FT:

6 $$E = F_0 \times {\boldsymbol{F}}\left( {E_0 \times F} \right)$$

here *F* represents the two-dimensional FT. Moreover, as with the other type of scalar diffraction, Fraunhofer diffraction in the far field can be expressed by $E = F_0 \times {\boldsymbol{F}}\left( {E_0} \right)$, which can be regarded as a special case of Fresnel diffraction passing through a converging lens. Therefore, scalar diffraction can be computed across the *xy*-plane using an FT-based approach.

Scalar diffraction can be used to effectively compute the complex amplitude distribution of many optical systems with a few approximations, as described above. However, it is known that the polarization components are changed due to large refractivity after passing through a high-NA non-paraxial system, and scalar diffraction is incapable of achieving proper results. The vectorial Debye diffraction integral, established by Richards and Wolf^21^, has to be adopted to analyze the complex EM field of each polarization component (Supplementary Information Section 1). The optical layout is shown in Fig. 1b.

Due to the refraction of the non-paraxial tight focusing system, the electric field components (polarization components $\overrightarrow e _s$ and $\overrightarrow e _p$) on the entrance pupil **P**_**e**_ are transformed into a spherical reference surface **P**_**r**_ ($\overrightarrow e _s$, $\overrightarrow e _{th}$, and $\overrightarrow e _r$). The transformation can be expressed in Cartesian coordinates as^20^:

7 $$\overrightarrow E _r = A_0\sqrt {\cos \theta } \times {\mathbf{M}} \,\times\, \overrightarrow E _i$$

**M** is the transform matrix of the polarization from the entrance surface to the converging spherical surface. $A_0\sqrt {\cos \theta }$ is the apodization factor accounting for the energy conservation. The propagation of the electric field from the reference surface **P**_**r**_ to the imaging point *p* (*x, y, z*) near the focus is expressed by the Debye integral:

8 $$\overrightarrow E = - \frac{{iC}}{\lambda }\mathop {\iint}\nolimits_{\!\Sigma} {\overrightarrow E _r} \times \exp \left[ {i\left( {k_zz - k_xx - k_yy} \right)} \right]{\mathrm{ }}d\Sigma$$

The definition of $\overrightarrow k _r$can be found in Supplementary Information Section 1. By performing the integration over the planar surface **P**_**e**_ instead of the surface **P**_**r**_ (Supplementary Information Section 2):

9 $$\overrightarrow E = - \frac{{iC}}{\lambda }\mathop {\iint}\nolimits_{\!\Omega} {\left[ {\overrightarrow E _r \times {{\exp \left( {ik_zz} \right)} / {\cos \theta }}} \right] \times \exp \left[ { - i\left( {k_xx + k_yy} \right)} \right]} {\mathrm{ }}dk_xdk_y$$

which can be rewritten in the form of an FT:

10 $$\overrightarrow E \left( {x,y,z} \right) = - \frac{{iC}}{\lambda }{\boldsymbol{F}}\left[ {\overrightarrow E _r \times {{\exp \left( {ik_zz} \right)} / {\cos \theta }}} \right] = - \frac{{iC}}{\lambda }{\boldsymbol{F}}\left[ {{\mathbf{M}} \times \overrightarrow E _i \times {{\exp \left( {ik_zz} \right)} / {\sqrt {\cos \theta } }}} \right]$$

In brief, both scalar diffraction and vector diffraction can be expressed by the FT. FFT algorithms in modern computer systems allow for fast and accurate calculations. The similarity between these two diffractions is obvious from a mathematical point of view: the vector diffraction integral is equivalent to the scalar Fraunhofer diffraction in the case of a low-NA optical system where 1/cos*θ* ≈ 1.

Although the FFT-based optical calculation is much faster than the direct integration method, it results in inevitable drawbacks: the resultant output field has a fixed transverse dimension and unchangeable sampling numbers determined by the dimension and sampling size of the input aperture for a given distance. The dimension of the output field is:

11 $$D_m = \frac{{\lambda d}}{{p_s}}$$

where *d* is the distance between the input aperture and output plane. *p*_*s*_ is the sampling size of the input aperture. The sampling numbers of the output plane are rigidly equivalent to those of the input aperture. The restriction is brought about by the intrinsic characteristic of the FT and greatly limits the flexibility in light propagation calculations. For example, the input aperture must be enormously expanded with the aid of the zero-padding approach when a small portion of the output plane is required with high resolution, which inevitably leads to a large increment of the computation time.

### Bluestein method to compute Fourier transform with arbitrary ROI and sampling resolution

Regarding mathematics, to achieve the required bandwidth and resolution in the frequency domain, the appropriate zero-padding operation is needed to extend the dimension of the original input sequence^15^. For most applications in laser manipulation and lithography, only a small fraction of the output field with high resolution is needed to obtain sufficient details, resulting in large amounts of zero-padding. This results in a severe waste of resources, as most of the results are discarded. The operation of the zero-padding inevitably increases the computation time and the demand for memory usage. Moreover, the resultant output region remains unchangeable, greatly limiting its potential in practical applications. Here, the Bluestein method is adopted to evaluate the scalar and vector diffraction calculations. The Bluestein method is an elegant method conceived by L. Bluestein^22^ and further generalized by L. Rabiner et al.^23^ that is capable of performing more general FTs at arbitrary frequencies as well as boosting the resolution over the full spectrum. The Bluestein method offers us a spectral zoom operation with high resolution and arbitrary bandwidth. This advantage is enabled by computing the z-transform along spiral contours in the *z*-plane for an input sequence (Supplementary Information Section 3 and Fig. S1). The computational complexity is *O*[(*M* + *N*)log_2_(*M* + *N*)], manifesting an FFT algorithm. The method is based on the z-transform along a spiral contour in the *z*-plane defined by *A* and *W*:

12 $$X\left[ m \right] = \left. {\mathop {\sum}\limits_{n = 0}^{N - 1} {x\left[ n \right] \times z^{ - n}} } \right|_{z = A \times W^{ - m}} = \mathop {\sum}\limits_{n = 0}^{N - 1} {x\left[ n \right] \times A^{ - n} \times W^{mn}}$$

here $m = \left[ {0, \cdots ,M - 1} \right]$. *M* is the length of the transform. *N* is the length of input sequence. *A* specifies the complex starting point of the *z*-plane spiral contour of interest, and *W* specifies the complex scalar describing the complex ratio between points along the contour. Note that the case of *A* = 1, *W* = exp(−*i*2*π/N*), and *M* = *N* corresponds to the discrete Fourier transform (DFT), which computes the z-transform along the unit circle with a finite duration. More generally, the method can be used to calculate the DFT between an arbitrary starting point *f*_*1*_ and ending point *f*_*2*_ (i.e., the tuneable frequency bandwidth relative to the total frequency range *f*_*s*_) with arbitrary sampling numbers *M*.

The practical implementations of the Bluestein method for enhanced DFT computation deserve additional comments. First, a 2D FT is needed for the computation of both scalar and vector diffraction. The Bluestein method should be adopted in both the column and the row dimensions to fulfill this requirement. Second, the Bluestein method internalizes padding of the input array with zeros at the tail. However, symmetric zero-padding around the input array is needed for the simulation of realistic optical systems. Third, an additional operation is needed to shift the zero-frequency component to the center of the array before and after the DFT to eliminate the high-frequency oscillation in the phase information. To address these issues, the definition of parameters *A* and *W* should be rearranged, and phase shifting factor *P*_shift_ should be included at the end of the calculation (see Supplementary Information Section 3 and Figs. S2–S4).

By performing these adjustments, the Bluestein method can be developed as a fast approach for light diffraction calculation with superior flexibility: it allows for the selection of arbitrary segments in the imaging plane with arbitrary resolution, providing competitive efficiency and flexibility over direct integration and the FFT methods.

### Fast numerical implementation of the Bluestein method in scalar Fresnel diffraction

**Fig. 2:**
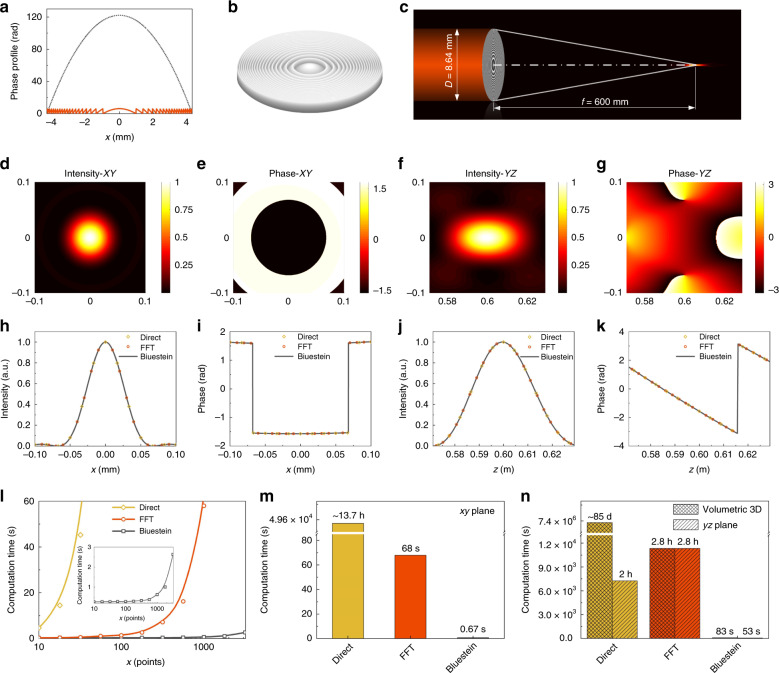
Scalar calculation of the converging spherical wave. **a** Phase profiles of the convex lens (gray line) and the corresponding phase plate (red line). **b** 3D rendered diagram of the phase plate. **c** Illustration of the optical setup. **d** Intensity and **e** phase distributions in the focal plane (*z* = 600 mm). **f** Intensity and **g** phase distributions in the longitudinal direction. **h**–**k** Line plots corresponding to (**d**–**g**), calculated using three different methods. **l** Dependence of the computation time on the number of sampling points in one dimension. An incident light field with sampling points of 1080 × 1080 and an interval of 8 μm (i.e., width of 8.64 mm) is fixed for each calculation (the same hereinafter unless otherwise specified). **m** Comparison of the computation time for the light field in the *xy*-plane using different methods. Here, the target region with a width of 0.2 mm is fixed with sampling points of 1080 × 1080. **n** Comparison of the computation time for the light field in volumetric three dimensions and the cross-sectional *yz*-plane using different methods. Here, 150 sliced layers are calculated

Figure 2 illustrates the scalar calculation with a paradigm of the converging spherical wave propagation, which is generated by a plane wave passing through a convex lens. The phase profile of the lens is shown in Fig. 2a, which is equivalent to the phase plate after being wrapped between 0 and 2*π* (Fig. 2b). The optical configuration is sketched in Fig. 2c, with the parameters *λ* = 800 nm, *f* = 600 mm, and *D* = 8.64 mm. Figure 2d, e shows the optical field distribution in the focal plane in terms of the intensity and phase. Figure 2f, g shows the cross-sectional intensity and phase distributions in the light propagation direction. The corresponding line plots of the intensity and phase are given in Fig. 2h–k. A comparison between the Bluestein method and traditional direct integration and FFT methods is also made, from which we can see excellent agreements. It is revealed that the Bluestein method can calculate the scalar light diffraction with high accuracy.

The Bluestein method has a superior advantage in the computation time cost over the direct integration and FFT methods. Due to the tedious point-by-point calculation method, the direct integration method is associated with two cycling loops, and the computation time increases drastically with the calculation points of the target plane (with a computational complexity of *O* (*M*^*2*^ × *N*^*2*^)). For the case of the FFT method, a zero-padding operation is needed to fulfill the requirement for the pre-set target sampling numbers, resulting in a rapid increase in computation time with the sampling points. As shown in Fig. 2l, with the increase in the sampling points along one coordinate axis, the Bluestein method exhibits its obvious superiority compared with the other two methods. This advantage makes the method particularly applicable to scenarios where large sampling points are needed, such as high-resolution microscopy. For the case in Fig. 2d, e, where the sampling points in the entrance pupil and output field are the same (*M* = *N* = 1080) and the ROI is 0.2 × 0.2 mm, the computational cost is ~13.7 h for the direct integration method, making it unsuitable for practical applications. For the FFT method, the computational cost is improved to 68 s, as shown in Fig. 2m. In comparison, the computation time is only 0.67 s using our proposed Bluestein method, which is 10^5^ and 10^2^ times less than those of the direct integration method and FFT method, respectively. The three-dimensional volumetric light field (Supplementary Information Fig. S5) can be reconstructed using cross-sectional light fields by calculating the lateral planes layer by layer. As depicted in Fig. 2n, the computation time for the direct method is excessively long to obtain the volumetric light field (~85 days). It takes 2 h to calculate the cross-sectional light field in the longitudinal *yz*-plane. By using the FFT method, the computational cost is the same (2.8 h) for both the volumetric and cross-sectional light fields because the ROI cannot be tuned due to the intrinsic characteristic of the FT. Owing to the fast computation property of the Bluestein method, calculation of the 3D optical field can be accomplished in <100 s. The efficiency enhancement is on the same order as that in the lateral *xy*-plane. More examples of scalar diffraction are given in Supplementary Information Section 4 and Fig. S6.

**Fig. 3:**
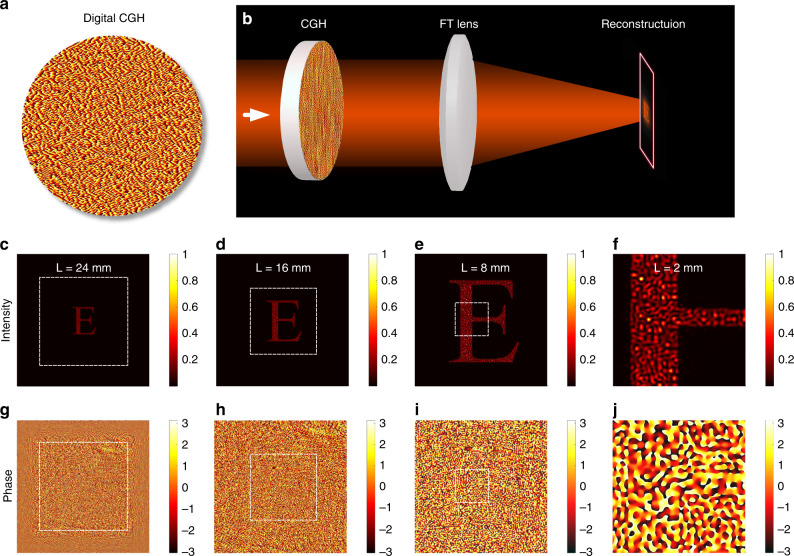
Scalar calculation of the CGH-modulated light wave. **a** Digital CGH derived from the GSW algorithm. **b** Optical setup for holographic reconstruction. Here the focal length of the FT lens is 600 mm. The dimension of the CGH is 8.64 mm (1080 × 1080 pixels), and the incident wavelength is 800 nm. **c**–**f** Calculated intensity distributions with changing ROIs. **g**–**j** Calculated phase distributions with changing ROIs. Here, sampling points are fixed at 1080 × 1080

In addition to the great improvement in computational efficiency, the Bluestein method has remarkable flexibility compared with the FFT method. That is, an arbitrary ROI can be defined with arbitrary resolution. This feature is illustrated by reconstructing a computer-generated hologram (CGH), as shown in Fig. 3. Evaluation of the light propagation after being modulated by a CGH is essential for predicting the performance of optical holographic tweezers^24^, holographic displays^25^, and laser holographic processing^26,27^. As shown in Fig. 3a, a CGH is generated by the weighted Gerchberg–Saxton (GSW) algorithm^28,29^. After FT by a converging FT lens, the designed pattern can be reconstructed (Fig. 3b). The process involves two scalar diffraction calculations: one is from the CGH to the FT lens, and the other is from the FT lens to the reconstruction plane. Figure 3c–f shows the intensity distributions with varying regions in the reconstruction plane and constant sampling points (1080 × 1080). Figure 3g–j shows the corresponding phase distributions. It is validated that the Bluestein method possesses fine flexibility compared with the FFT method.

### Fast numerical calculation of the vectorial Debye diffraction

**Fig. 4:**
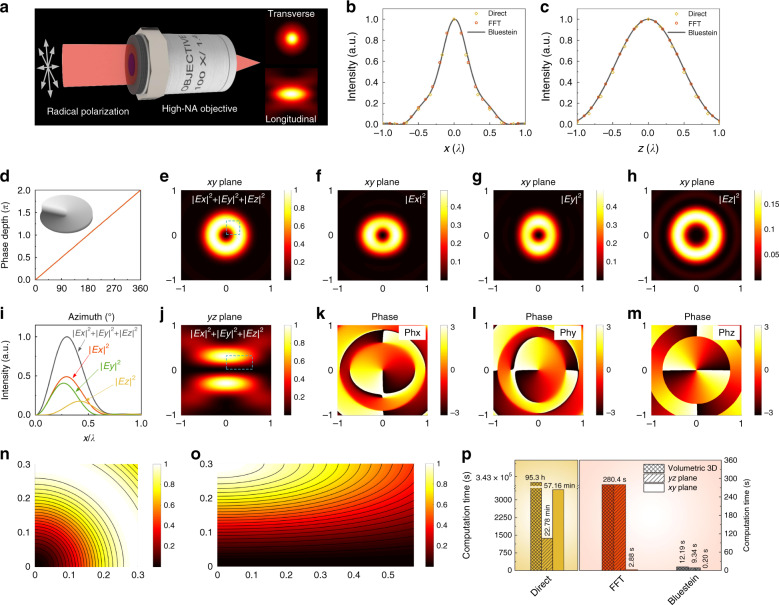
Vector diffraction of light with a high-NA objective. **a** Radially polarized light focused by an aplanatic objective (NA: 1.4). Insets: resultant intensity profiles in the transverse and longitudinal planes. **b**, **c** Line plots of the intensity in the transverse and longitudinal directions calculated using different methods. **d** Spiral phase plate and the phase depth distribution with the azimuthal angle. **e** Doughnut-shaped intensity in the focal plane. **f**–**h** Intensity distributions of different polarization components along the *x*, *y*, and *z* directions, respectively. **i** Line plots of the intensity profile of different polarization components in the focal plane. **j** Intensity distribution in the longitudinal plane. **k**–**m** Phase distributions of different polarization components. **n**, **o** Enlarged partial intensity distribution as labeled in (**e**, **j**), respectively. **p** Comparison of computation time for vector diffraction calculation in the *xy*-plane, *yz*-plane, and volumetric three dimensions

The vectorial nature of light is essential for optical systems with a high-NA aperture or specific polarization, such as radial and azimuthal polarizations^30,31^. Figure 4a illustrates the focusing of radially polarized light by a high-NA aplanatic objective (NA: 1.4). By using the proposed Bluestein method in the vectorial Debye–Wolf integral, the light field distribution near the focus can be rapidly calculated (insets of Fig. 4a). The results are consistent with those computed by direct integration and the FFT methods, as reflected by the line plots of the light intensity along the transverse and longitudinal directions in Fig. 4b, c.

The optical vortex generated by a spiral phase plate (Fig. 4d), in cooperation with circular polarization, plays a key role in super-resolution stimulated emission depletion microscopy^32^ and nano-lithography^33^. A doughnut-shaped focus profile with a dark center is used as the depletion beam to eliminate fluorescence or polymerization. Figure 4e, j shows the optical intensity profiles of the optical vortex in the lateral *xy* and longitudinal *yz*-planes, respectively. An engineered focus with a symmetric doughnut shape can be generated. Moreover, the light components in different polarizations can be obtained efficiently using our Bluestein method, as shown in Fig. 4f–i, k–m. It can be seen that all the light components have dark central intensities close to zero and the spiral phase. The light in the transverse polarizations is dominant over the longitudinal polarization. The Bluestein method also endows the vectorial calculation with high flexibility compared with the traditional FFT approach. Figure 4n, o shows the enlarged intensity profiles in the ROIs labeled in Fig. 4f, g, respectively. Another example of the usage of the Bluestein method for vector diffraction is shown in Supplementary Information Section 5 and Fig. S7. The optical information in the arbitrary ROIs can be investigated in detail without increasing the computational cost, making the Bluestein method advantageous in evaluating localized high-resolution light distributions for the application of microscopy and photolithography.

For the computation time, the Bluestein method also exhibits great superiority. Here, we consider the calculation from the entrance pupil with ~10^5^ sampling points to the exit pupil with the same points in the *xy*-plane, and 100 layers along the optical axis are calculated for volumetric and cross-sectional light distributions in the *yz*-plane. As shown in Fig. 4p, the direct method requires 57.16 min to calculate the lateral light field. 95.3 h is needed for the volumetric 3D light field distribution, and 22.78 min is needed for the sliced *yz*-plane. An acceptable time (2.88 s) is needed for the FFT method to calculate the *xy*-plane. However, an impractical 280.4 s is needed to obtain the light distribution in the volumetric three dimensions and the two-dimensional *yz*-plane. In contrast, only 0.2 s is consumed by the Bluestein method for calculation in the *xy*-plane. Moreover, only 9.34 and 12.19 s is needed to achieve the 2D cross-sectional and 3D volumetric light fields. Note that the computation time increases much more quickly with the sampling numbers of the ROI for the direct method and FFT method than for the Bluestein method, e.g., more than 10 days are needed for the direct method and 126.5 s is needed for the FFT method to acquire transverse light distributions in the *xy*-plane when the number of sampling points increases to ~10^6^ (1080 × 1080), while only 1.78 s is needed for the Bluestein method, which is five orders of magnitude less than that needed for the direct method and 10^2^ times less than that for the FFT method.

### Full-path optical calculation with superior flexibility and efficiency

**Fig. 5:**
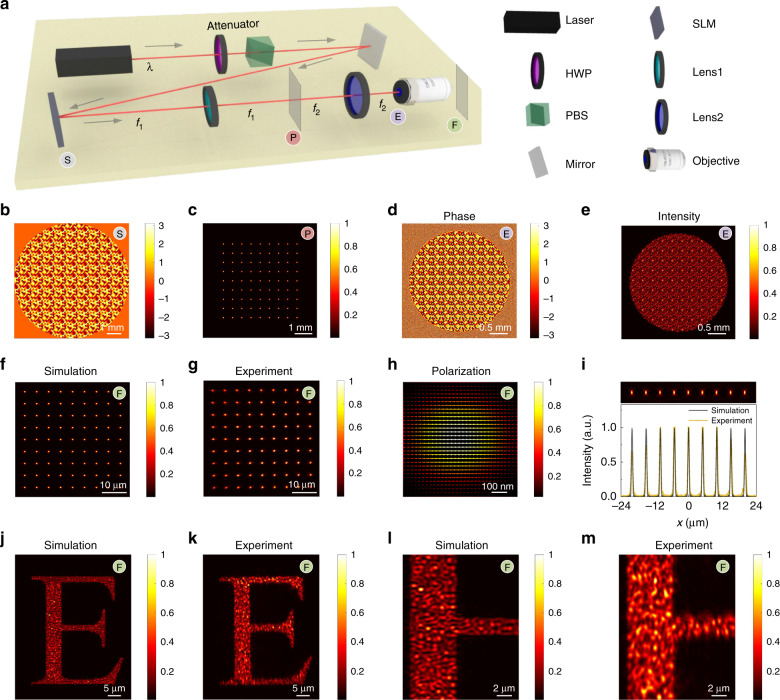
Full-path calculation of a representative optical system. **a** Sketch of the optical system. *S*: the plane on the panel of the SLM. *P*: the focal plane of Lens 1. *E*: the entrance pupil of the objective. *F*: the focal plane of the objective. (**b**) CGH displayed on the SLM for the generation of a 9 × 9 foci array. **c** The foci array on the focal plane of Lens 1 (*P-*plane). **d** Phase distribution and **e** intensity distribution on the entrance pupil of the objective (*E*-plane). **f** Simulated and **g** measured multi-foci array generated on the focal plane of the objective (*F*-plane). **h** Enlarged intensity profile of a single focal spot in the array. The arrows indicate the polarization directions. **i** Longitudinal intensity profile and corresponding line plot of the foci array. **j** Simulated and **k** measured intensity distribution on the *F*-plane when the CGH for the generation of the pattern “E” is encoded on the SLM. **l**, **m** Enlarged intensity profiles of the pattern corresponding to (**j**) and (**k**) with the same sampling points as in (**i**)

As discussed above, both the scalar and vector diffraction can be efficiently calculated by the Bluestein method. Based on this, the full-path optical calculation and tracing can be performed with high flexibility and efficiency. Figure 5a illustrates a representative optical layout for laser holographic processing and holographic manipulation. This setup can be further adopted for two-photon scanning confocal microscopy. Here, a phase-only spatial light modulator (SLM, Holoeye Pluto NIR-II, resolution: 1920 × 1080) is used to modulate the wavefront of the laser by loading a predesigned CGH. A combination of a half-wave plate and polarization beam splitter is utilized to attenuate the laser power. A 4*f* configuration consisting of Lens 1 (*f* = 600 mm) and Lens 2 (*f* = 200 mm) is placed between the SLM and aplanatic objective (100×, NA: 1.4). It is a typical optical system involving both scalar diffraction and vector diffraction during light propagation.

First, we simulate the multi-foci optical system, which can be used for holographic tweezers, laser parallel processing and data recording. Figure 5b is the corresponding CGH for the generation of a 9 × 9 multi-foci array. A linearly polarized femtosecond laser (800 nm, emitted from Chameleon Vision-S, Coherent) is modulated by the CGH. After the FT of Lens 1, a multi-foci array is generated (Fig. 5c). At the back of the objective, the phase and intensity distributions are retrieved as shown in Fig. 5d, e. The phase profile closely resembles the CGH, validating the accuracy of the Bluestein-enabled calculation of scalar diffraction. The light beam is slightly smaller than the size of the entrance pupil of the objective, ensuring that the phase-modulated beam can be fully transformed by the objective. In the focal plane of the objective, a diffraction-limited 9 × 9 multi-foci array is generated (Fig. 5f). The full-path calculation can be accomplished with high efficiency in <4 s. The experimentally measured multi-foci intensity (Fig. 5g) agrees well with the simulation. With the help of the highly flexible Bluestein method, a detailed analysis of a single focal spot is enabled, as shown in Fig. 5h, revealing that a Gaussian focus is generated with linear polarization. The light field in the longitudinal section can be readily computed, and the spatial uniformity can be investigated (Fig. 5i).

Another universal example is given in Fig. 5j–m, where a CGH is encoded on the SLM to generate a pattern as discussed in Fig. 3. By using the Bluestein full-path calculation method, the light field of the desired pattern can be simulated in the focal plane of the objective (Fig. 5j), consistent with the experimental result (Fig. 5k). By taking advantage of the high flexibility of the Bluestein method, a magnified image of an arbitrary ROI can be calculated with arbitrary resolution and good accuracy in comparison with the experimental result, as shown in Fig. 5l, k. Another example of the usage of the Bluestein method for vector diffraction is shown in Supplementary Information Section 6 and Fig. S8. In brief, full-path light tracing of the entire optical system can be accomplished by the Bluestein method with high efficiency and flexibility, unfolding its capacities in the real-time prediction and evaluation of optical performance in advanced microscopy, laser manipulation, and photolithography.

## Discussion

The proposed Bluestein-based method provides a fundamental improvement in optical diffraction calculations. The advantages of the method lie in the following three aspects. First, the computation method for light diffraction is superfast, allowing for the real-time prediction of light field propagation for diverse implementations. Second, the method has great flexibility, without loss of accuracy and efficiency. The desired ROI can be freely chosen, and the sampling numbers can be arbitrarily tuned. Third, the method shows good universality. It suits all diffraction conditions, such as phase modulation, amplitude filtering, polarization conversion, and focusing transform. In particular, this method facilitates the simulation and optimal design of metasurfaces^34–36^, as exemplified in Supplementary Information Section 7 and Fig. S9. Both scalar and vector diffraction can be computed using this method, making this method promising for full-path propagation evaluation in broad applications of optical microscopy, lithography, and optical manipulation.

In addition, the applicability of this method needs to be explicated for realistic implementations. First, some approximation conditions are assumed for both vector and scalar diffraction. For vector diffraction, the lens is assumed to obey Abbe’s sine condition. For scalar diffraction, the Fresnel approximation should be valid. It is worth noting that Fraunhofer diffraction can also be implemented using the Bluestein method with slight modification. Second, it is important to take stringent precautions against aliasing effects. When the diffraction distance of scalar diffraction is too small or the focal shift of vector diffraction is too long, obvious aliasing is likely to occur because the sampling condition no longer satisfies the Nyquist sampling condition.

In summary, an efficient calculation method is developed to evaluate light diffraction with high flexibility and efficiency. First, a set of mathematical preliminaries is given to express the scalar and vector diffraction integrals in the form of an FT and then unified using the Bluestein method. Examples for both scalar and vector diffraction are demonstrated to reveal that the computational efficiency and flexibility are greatly improved. Calculation of the light field is realized at the sub-second time level compared with several minutes using the FFT method or hours using the direct integration method. Full-path light tracing is finally demonstrated using the Bluestein method. This method holds great potential not only in the fast prediction of numerous optical systems but also in the realm of signal processing for acoustic and other communication waves.

## Materials and methods

### Computational environment

All the calculations are performed on a personal laptop with an Intel processor I5 2.50 GHz and 8 GB of memory, running the Windows 10 Professional operating system. The code is written, compiled and run in the MATLAB R2019a software. All the comparison studies on efficiency are performed in the same computational environment.

### Laser holographic system

The laser holographic system consists of a Ti:sapphire femtosecond laser oscillator (Chameleon Vision-S, Coherent) with a central wavelength of 800 nm, a repetition rate of 80 MHz, and a pulse width of 75 fs. A phase-only reflective liquid crystal SLM (Pluto NIR-2, Holoeye) is utilized for the phase modulation, which features a 1920 × 1080 resolution and a 8 μm pixel pitch. In the experiment, only the central portion of the SLM with 1080 × 1080 pixels is used to modulate the wavefront, while the other pixels are set to zero. A phase hologram pattern with 256 different shades of gray is loaded onto the SLM, corresponding to the phase modulation depth from 0 to 2*π*. A CCD camera is used to capture the light field distribution.

## Supplementary information

Supplementary Information
