# Supplementary material for: Efficient full-path optical calculation of scalar and vector diffraction using the Bluestein method
Source: Light Sci Appl. 2020 Jul 13;9:119. doi: 10.1038/s41377-020-00362-z (PMC7359032; doi:10.1038/s41377-020-00362-z)
Supplement: Supplementary file 1 — Supplementary Information [file 41377_2020_362_MOESM1_ESM.docx]

Supplementary Information for

Efficient full-path optical calculation of scalar and vector diffraction using the Bluestein method

Yanlei Hu,^1,2^ Zhongyu Wang,^1^ Xuewen Wang,^3^ Shengyun Ji,^1^ Chenchu Zhang,^4^ Jiawen Li,^1*^ Wulin Zhu,^1^ Dong Wu,^1*^ Jiaru Chu^1^

^1^CAS Key Laboratory of Mechanical Behavior and Design of Materials, Key Laboratory of Precision Scientific Instrumentation of Anhui Higher Education Institutes, Department of Precision Machinery and Precision Instrumentation, University of Science and Technology of China, Hefei, 230026 China

^2^Department of Mechanical Engineering and Department of Civil and Environmental Engineering, Massachusetts Institute of Technology, Cambridge, Massachusetts 02139, United States

^3^State Key Laboratory of Advanced Technology for Materials Synthesis and Processing, International School of Materials Science and Engineering, Wuhan University of Technology, Wuhan 430070, China

^4^Institute of Industry and Equipment Technology, Hefei University of Technology, Hefei, 230009 China

*jwl@ustc.edu.cn

*dongwu@ustc.edu.cn

Section 1. Derivation of the direct numerical integration of vectorial Debye diffraction

Here we present the derivation of the direct integration method of vector Debye diffraction. From Figure 1b, we can derive that:

(S1)

Therefore,

$\vec{E}(x,y,z)=-\frac{iC}{\lambda}\iint_{\Sigma} \vec{E}_{r}\cdot\exp\left[i\left(k_{z}z-k_{x}x-k_{y}y\right)\right]d\Sigma$ (S2)

(S3)

(S4)

Here,

(S5)

In the imaging plane, we use the cylindrical coordinates (*r_2_**, φ_2_, z*) to replace the Cartesian coordinates (*x, y, z*):

(S6)

(S7)

Therefore,

(S8)

Section 2. Derivation of the FFT method of vectorial Debye diffraction

According to Eq. S3, the electric field can be written in the form:

(S9)

Because $\theta=\mathrm{asin}(\frac{\sqrt{k_{x}^{2}+k_{y}^{2}}}{kn})$, and . The Jacobian matrix for the conversion of coordinates from (*θ, φ*) to (*k_x_, k_y_*) is:

(S10)

Therefore,

$\begin{matrix} \vec{E}=-\frac{iC}{\lambda}\int_{0}^{\theta_{\max}} \int_{0}^{2\pi} \vec{E}_{r}\cdot\exp\left(ik_{z}z\right)/\cos\theta\\ \cdot\exp\left[-i\left(k_{x}x+k_{y}y\right)\right]dk_{x}dk_{y} \end{matrix}$ (S11)

(S12)

Section 3. Flexible Fast Fourier Transform using the Bluestein method:

As illustrated in Equation 12 in the main text, The Bluestein method computes the z-transform of the signal with finite duration along a general spiral contour in the z-plane by assigning the coefficient *z* as$z=A\cdot W^{-m}$. In order to better understand the spectral zoom operation of this method, all one needs to know is that *A* is the complex starting point,$A=A_{0}\cdot\exp\left(i\theta_{0}\right)$, *W* is a complex scalar describing the complex ratio between neighboring points on the contour (point spacing), , and *M* is the length of the transform. *A_0_* and *θ_0_* are the radius length and phase angle of the complex starting point, respectively. *W_0_* is the radius variance ratio of the spiral contour and *φ_0_* represents the angular spacing between two adjacent sampling points. Three typical cases are discussed below to better understand the ability of Bluestein method.

**Case 1:** when *A_0_*=1, *θ_0_*=0, *W_0_*=1, , and *M*=*N*, therefore $z=\exp\left(i\frac{2m\pi}{N}\right)$, Equation 12 in the main text corresponds to ordinary non-tunable DFT:

$X\left[m\right]=\sum_{n=0}^{N-1} x\left[n\right]\cdot\exp\left(-i\frac{2\pi mn}{N}\right)$ **(S13)**

In the view of *z* plane, the DFT is evaluated along a unit circle starting 0 thru 2π with equal spacing (Figure S1a), which is equivalent to the total frequency spectrum.

**Case 2:** when *A_0_*=1, and *W_0_*=1, Equation 12 corresponds to the flexible DFT as discussed in this work with arbitrary starting point, arbitrary end point and arbitrary sampling spacing. It can be visualized in z plane as an arc of the unit circle (Figure S1b). This is equivalent to a subsection of the total frequency spectrum.

**Case 3:** when *W_0_*≠1, general Bluestein FFT is achieved with a spiral contour in z plane with arbitrary starting point, end point and sampling spacing (Figure S1c). *W_0_* > 1.0 and *W_0_* < 1.0 correspond to the cases that contour spirals in and out from the unit circle, respectively.

In this work, we mainly focused on the case 2, where flexible and high-efficiency DFT can be realized using Bluestein method and further applied in the domain of diffractive optics. In other words, we limit the z-transform to the region of the unit circle. Flexible DFT can be achieved with arbitrary starting point, arbitrary length and arbitrary sampling numbers.


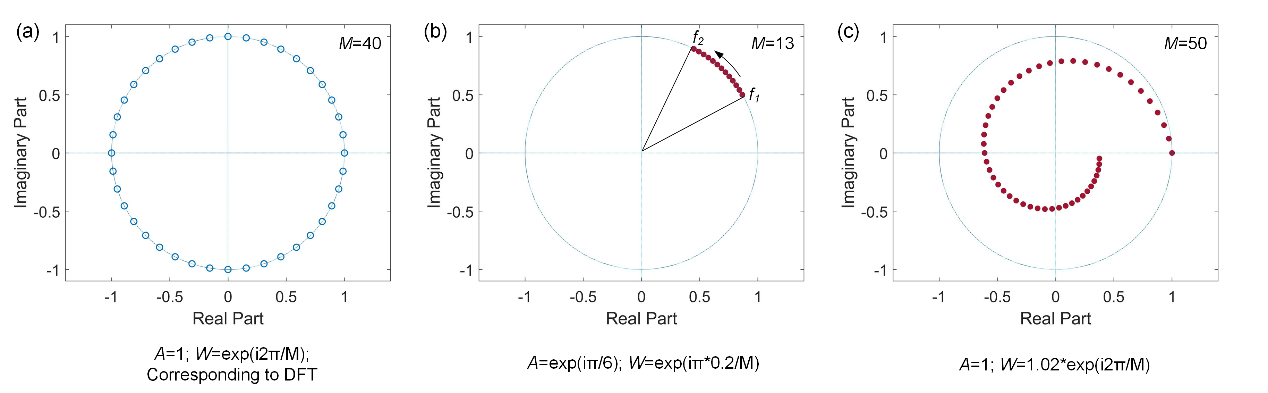


Figure S1. Representation of z-plane contours for different parameters *A*, *W* and *M*

Note that the build-in Bluestein method in MATLAB (chirp-z transform CZT) is carried out by using the circular convolution where zero-padding is performed at the tail of the input array. However, in practical applications for optical simulation, symmetric zero-padding around the input array is needed to locate the information of use in the center of the plane (Figure S2). In order to rectify the change, a positional shift on the result is needed:

$M_{shift1}=\frac{1}{2}\left(\frac{M\cdot f_{s}}{f_{2}-f_{1}}-N\right)$ (S14)


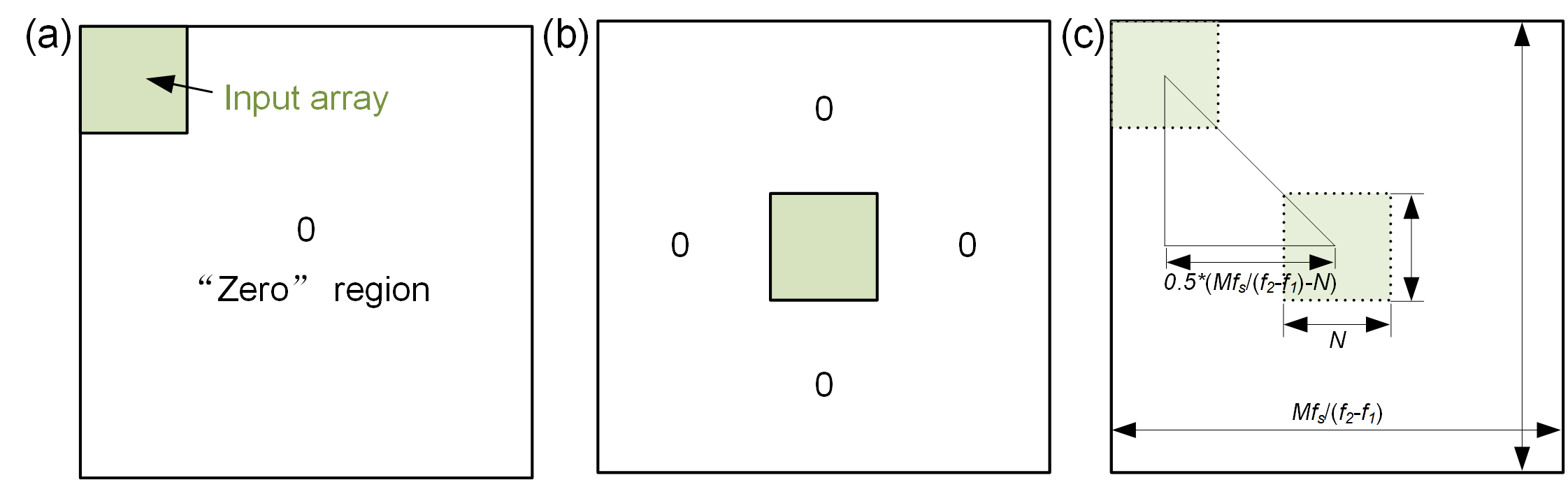


Figure S2. Diagram of the two-dimensional zero-padding in different ways. (a) Zero-padding in the tail of each dimensions, making the input array located in the top-left corner of the extended array. (b) Symmetric zero-padding, making the input array located in the center of the extended array. (c) Positional shift between these two zero-padding approaches.

In addition, direct DFT results in high-frequency oscillation in the phase information because the zero-frequency component is in the top-left corner of the resultant spectrum matrix (Figure S3 and Figure S4). Therefore, another positional shift is needed to move the zero-frequency component to the center of the array before and after DFT (corresponding to the “fftshift” command in the MATLAB software environment). To this end, the sign of *A* is inversed and another corresponding positional shift can be obtained:

$A=-\exp\left(i2\pi\frac{f_{1}}{f_{s}}\right)$ **(S15)**

$W=\exp\left(-i2\pi\frac{f_{2}-f_{1}}{Mf_{s}}\right)$ **(S16)**

$M_{shift2}=-\frac{1}{2}\frac{M\cdot f_{S}}{f_{2}-f_{1}}$ (S17)

Therefore, the total positional shift is:

$M_{shift}=-\frac{N}{2}$ (S18)

In order to compensate the positional shift in the object plane, a phase shift in the imaging plane is obtained:

$\begin{matrix} P_{shift}=\exp\left[-i2\pi\cdot M_{shift}\cdot\frac{m\left(f_{2}-f_{1}\right)\text{+}Mf_{1}}{Mf_{s}}\right] \\ =\exp\left[i\pi\cdot N\cdot\frac{m\left(f_{2}-f_{1}\right)\text{+}Mf_{1}}{Mf_{s}}\right] \end{matrix}$ (S19)

Example of algorithm codes as free open-source software can be found at <https://github.com/yanleihu/Bluestein-Method> to help ones to use this highly-efficient method conveniently.


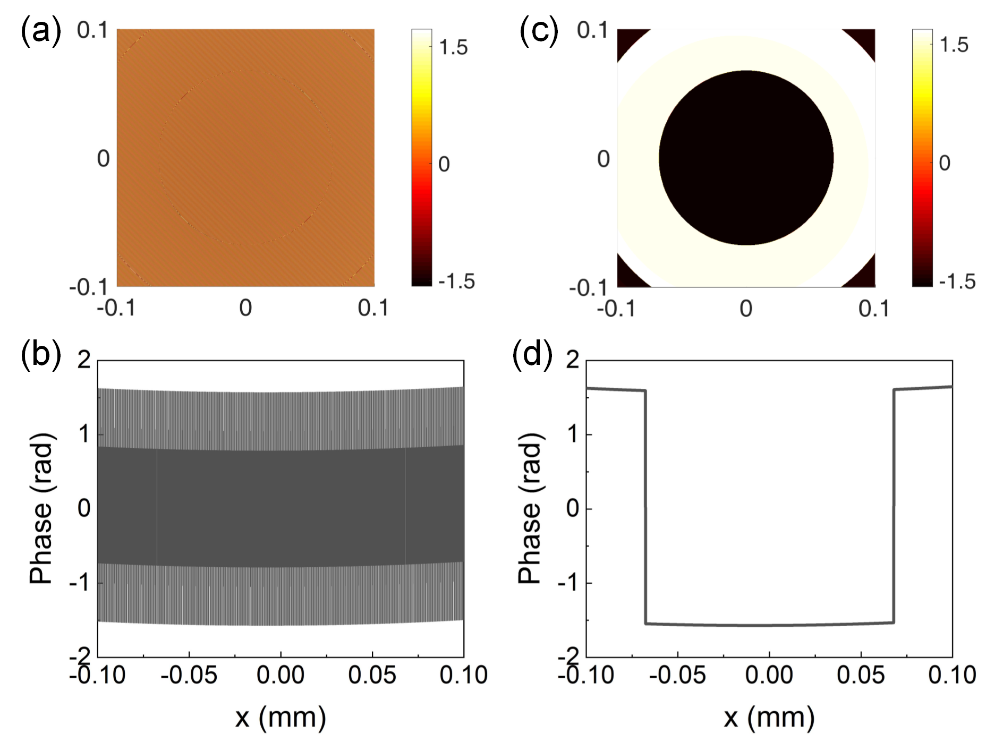


Figure S3. The phase retrieval results in the scalar diffraction (a-b) without zero-frequency component movement and (c-d) with zero-frequency component movement to the center of the array. The optical parameters are the same as these in Figure 2 in the main text.


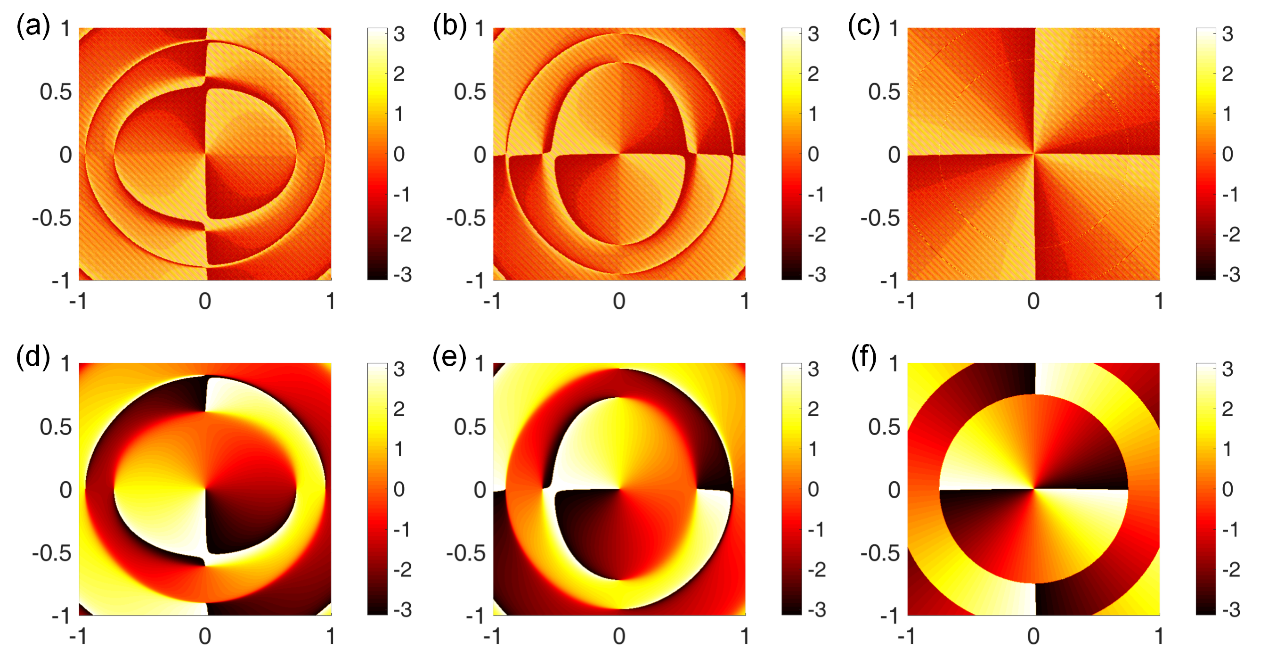


Figure S4. The phase retrieval results in the vector diffraction (a-b) without zero-frequency component movement and (c-d) with zero-frequency component movement to the center of the array. The optical parameters are the same as these in Figure 4 in the main text.


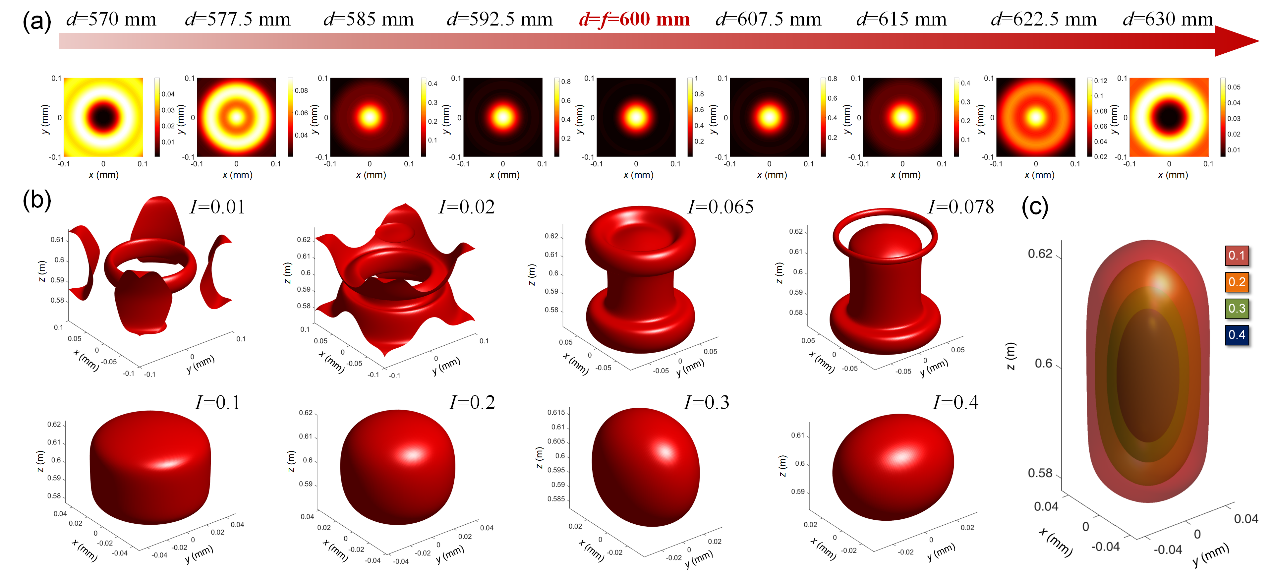


Figure S5. Volumetric light calculation using the Bluestein method. (a) Calculation of the cross-sectional light intensity distribution along the propagation with varying distances. The condition is the same as that in Figure 2 where the focal length of the lens is *f*=600 mm. (b) Contour profiles (isosurface) of the focus field with different intensity values: 0.01, 0.02, 0.065, 0.078, 0.1, 0.2, 0.3 and 0.4. The light intensity is normalized. (c) Volumetric 3D light field. Different colors represent different intensity value (after normalization). Note that the lengths of *xyz* axes in (b) and (c) are not to scale.

Section 4. Another example of efficient scalar diffraction calculation: zero-order Bessel beam

It is known that the non-diffracting Bessel beam characterizes a long needle of light intensity and can be generated using an axicon lens^1,2^. Here the generation and propagation of zero-order Bessel beam is investigation using our Bluestein-based scalar diffraction calculation method, as shown in Figure S6. The distinct features of light intensity and phase distribution of Bessel beam can be revealed in the calculation.


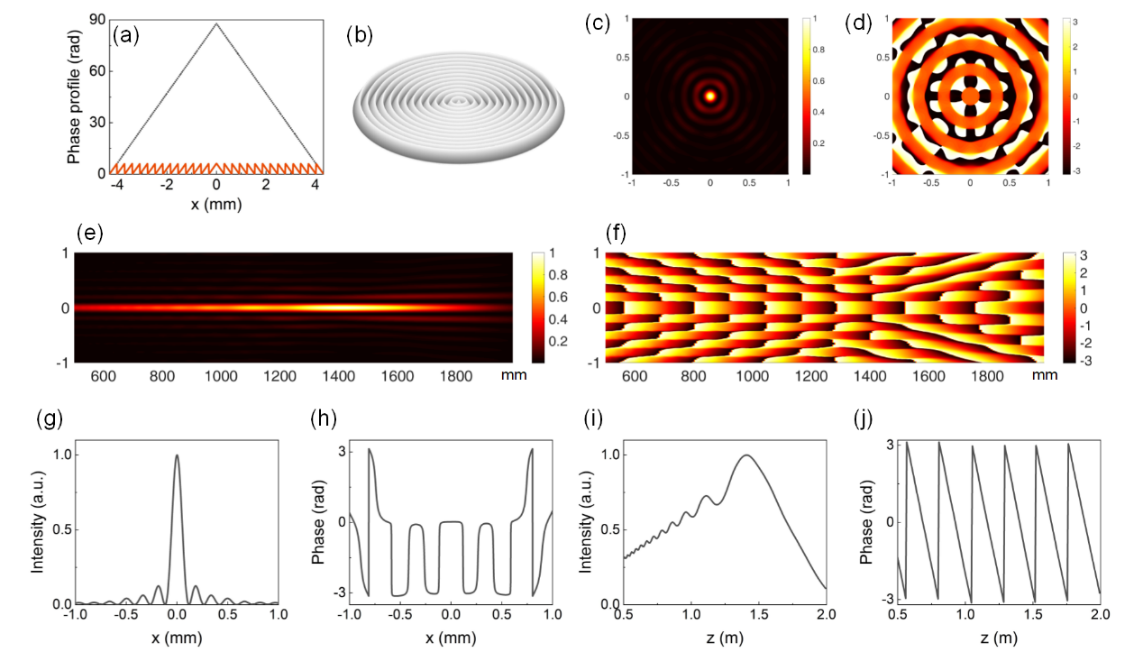


Figure S6. Another example of scalar calculation of the non-diffracting Bessel beam. (a) Phase profiles of the axicon lens and corresponding phase plate for generating Bessel beam. (b) 3D rendered diagram of the phase plate. (c) Intensity and (d) phase distributions in the plane *z*=1400 mm. (e) Intensity and (f) phase distributions in the longitudinal direction. (g-h) Line plots of intensity and phase profiles in transverse plane corresponding to (c-d). (i-j) Line plots of intensity and phase profiles in longitudinal plane corresponding to (i-j).

Section 5. Another example of efficient vector diffraction calculation: generation of long needle of longitudinally polarized light

It has been reported that pure longitudinal light bean with sub-diffraction beam size can be created by combining annular binary optics and radical polarization^3^. In order to verify the ability of the Bluestein vector diffraction calculation, we perform the calculation by adopting the same parameters in Ref. 3: there are four key diameters (*r_1_*=0.091; *r_2_*=0.391; *r_3_*=0.592; *r_4_*=0.768) to divide the binary phase plate into five regions (Figures S7 a and S7b). The light is focused by an objective (NA:0.95). It can be seen from Figures S7 (c-h) that non-diffracting longitudinal polarized beam is generated with a 0.43*λ* beam size and ~4*λ* propagating distance. The longitudinal component plays a dominant role and the radical components in *x* and *y* directions can be neglected.


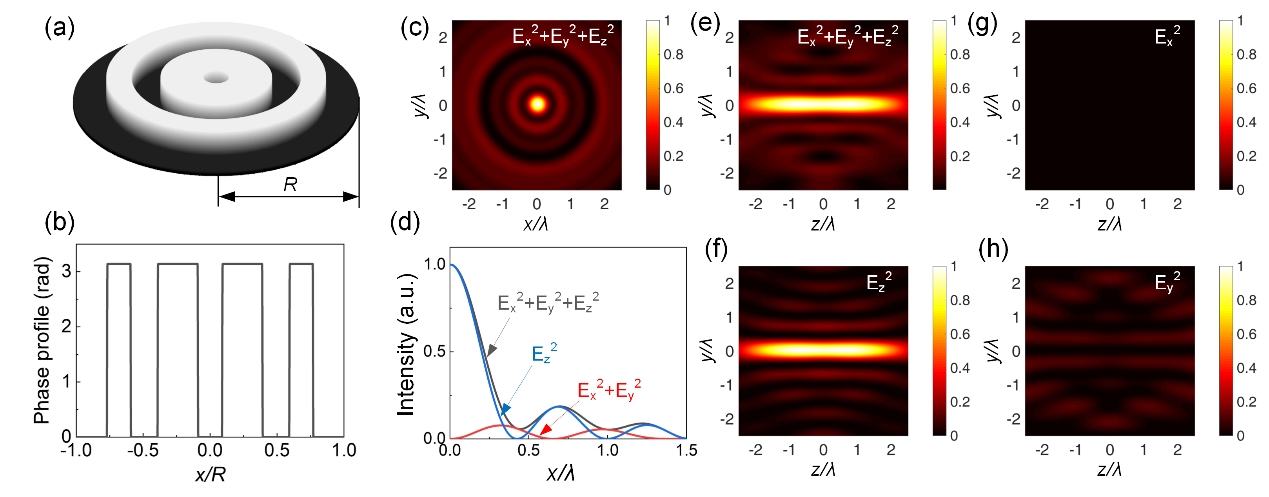


Figure S7. Vector diffraction to create a needle of longitudinally polarized light using binary phase plate. (a) The annular binary phase plate and (b) the phase profile in radial direction. (c) Transverse light intensity field in the focal region and (d) line plots of each polarization components. (e) Total intensity field on the *yz* cross-section. (f-h) Corresponding components in *z*, *x* and *y* directions.

Section 6. Efficient full-path calculation on the propagation of optical vortex

Another example on the full-path propagation of optical vortex in the scalar-vector hybrid optical system is given. The optical vortex is generated with a spiral phase hologram with three-fold periodicity and the doughnut-shaped light field with larger dark center (compared with that in Figure 4) is produced in the vector light field. Right-handed circular polarization is applied here for generation of optical vortex.


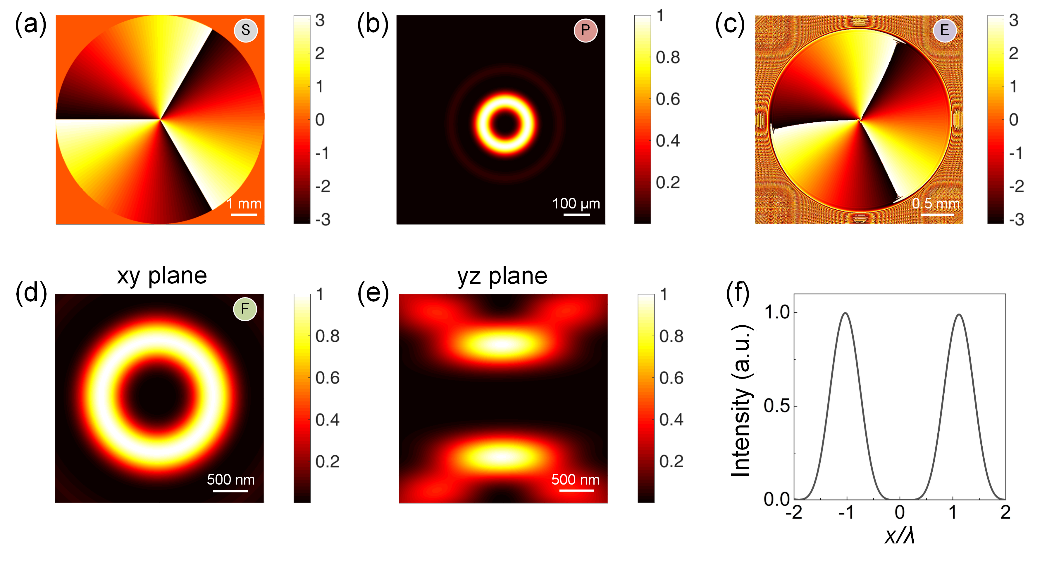


Figure S8. Full-path calculation of the propagation of optical vortex^4^. (a) A spiral phase hologram is encoded on the SLM to generate the optical vortex with a topological charge *l*=+3. (b) The doughnut-shaped optical vortex on the focal plane of Lens1 (P plane). (c) Phase distribution on the entrance pupil of the objective (E plane). (d) The optical vortex generated on the focal plane of the objective (F plane). (e) Longitudinal intensity profile. (f) Line plot of the intensity of optical vortex in the radial direction.

Section 7. Bluestein method for simulation and design of metasurfaces

The proposed Bluestein method is able to calculate the theoretical optical field generated by metasurfaces. For example, several kinds of planar metasurfaces including metalens and vortex generator have been innovatively developed. As shown in Figures S9 (a-f), the focal performance of the illustrious metalenses can be simulated using the Bluestein method. Moreover, we can also simulate the condition with fabrication errors as shown in Figure R3. The errors can be tailorable according to the realistic processing condition. Here certain random errors are considered in the metalenses in order to simulate the fabrication errors. The influence of fabrication errors on the focal performance can be readily investigated. In this way, one can study the fabrication tolerance of the nanostructures and thus optimize the devices design and fabrication process.


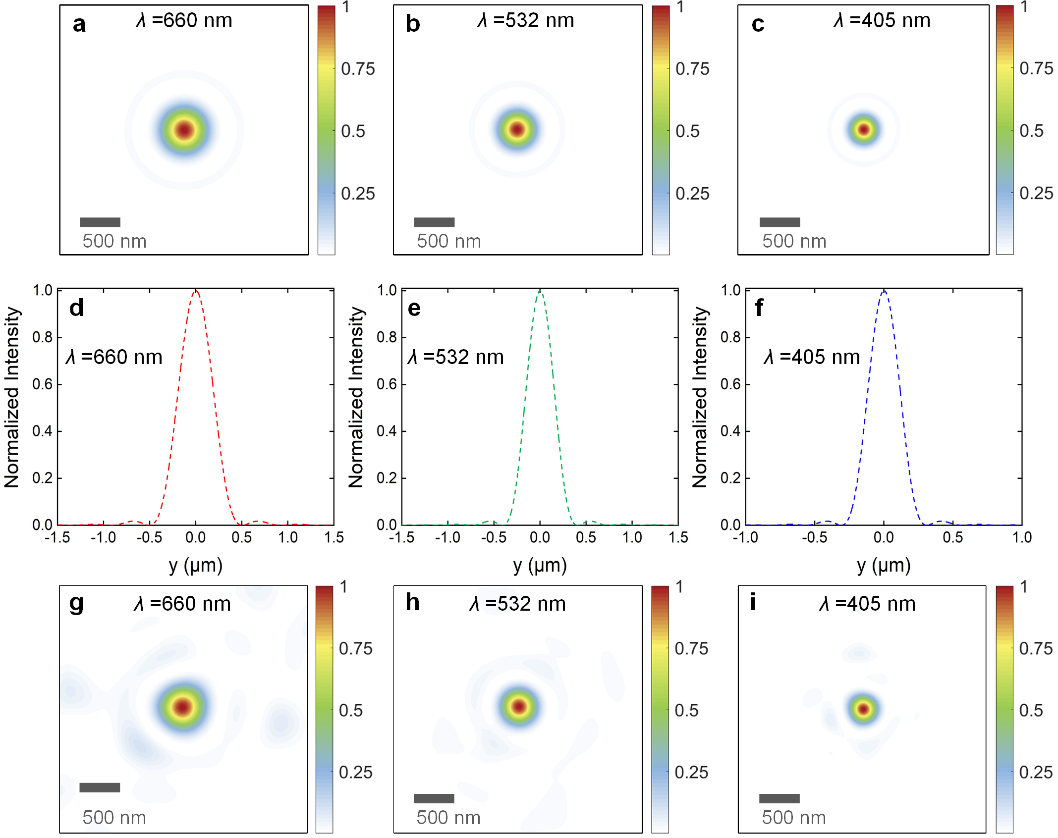


Figure S9. Diffraction-limited focal spots of three metalenses designed for different wavelength. (a-c) Theoretical simulations of the focal spots using the proposed Bluestein method on the focal spots intensity distributions of three metalenses designed for 660 nm, 532 nm, and 405 nm, respectively. (d-f) Line plots of the focal intensity of three metalenses. (g-i) Simulated results by considering the fabrication errors of the metalenses. Here random errors are assumed in the calculation, which can be flexibly tailored according to the realistic fabrication condition.

References

1 Planchon, T. A. *et al.* Rapid three-dimensional isotropic imaging of living cells using Bessel beam plane illumination. *Nature Methods* **8**, 417 (2011).

2 Durnin, J., Miceli, J. & Eberly, J. H. Comparison of Bessel and Gaussian beams. *Optics Letters* **13**, 79-80 (1988).

3 Wang, H., Shi, L., Lukyanchuk, B., Sheppard, C. & Chong, C. T. Creation of a needle of longitudinally polarized light in vacuum using binary optics. *Nature Photonics* **2**, 501 (2008).

4 <https://github.com/yanleihu/Bluestein-Method>.
